# The effects of working memory training on decision-making in collegiate basketball referees: a randomized controlled trial

**DOI:** 10.3389/fpsyg.2026.1946720

**Published:** 2026-09-23

**Authors:** Pengfei Kang, Heng Zhang, Qiulin Wang

**Affiliations:** 1 Department of Physical Education, Nanjing University of Finance & Economics, Nanjing, Jiangsu, China; 2 Taicang Experimental High School, Taicang, Jiangsu, China; 3 College of Physical Education, Yangzhou University, Yangzhou, Jiangsu, China

**Keywords:** collegiate basketball officials, decision-making, working memory, working memory capacity, working memory enhancement

## Abstract

**Background:**

Collegiate basketball referees necessitate swift information processing and precise decision-making under duress, with working memory serving as a fundamental cognitive function. Investigations into cognitive training for referees are still few.

**Methods:**

This randomised controlled trial investigated the impact of an 8-week working memory training program on decision-making among 28 collegiate basketball referees (ages 19–22), who were allocated to either an experimental group (*n* = 14) or a control group (*n* = 14). The accuracy of decision-making, response time, and working memory capacity were evaluated.

**Results:**

The experimental group exhibited notable enhancements in decision-making accuracy, especially with cognitive decisions; diminished response times; and augmented working memory capacity.

**Conclusion:**

Working memory training improves cognitive efficiency in collegiate basketball referees with limited officiating experience, facilitating quicker and more precise officiating decisions under laboratory static-image testing conditions. These observed medium-sized effects are sample-specific and should not be generalized to professional elite referees. Targeted cognitive training may function as an effective strategy to enhance officiating performance for this specific population.

## 1 Introduction

In basketball, referees serve as essential enforcers of the rules (Wang et al., 2021). Their decisions directly affect the game’s outcome and greatly influence the athletes’ performance and the competition’s overall ranking. In team sports, officiating requires applying the rules to available situational information and selecting an appropriate action (Kittel et al., 2025). The referees’ judgements profoundly influence the game’s dynamics, enjoyment, and equity. Effective officiating preserves order in the game and guarantees its seamless advancement (Ding et al., 2021). In-depth study of refereeing judgements can increase referees’ professional skills, allowing for a deeper comprehension of the rules, improved competence, and the ability to maintain composure and objectivity in high-pressure situations. In China, the majority of collegiate basketball referees undergo initial training at diverse sports colleges or physical education faculties within teacher training universities (Wei and Li, 2009). College basketball referees from sports education programs are defined by their status as students, little practical officiating experience, and a nascent comprehension of the rules and the dynamics of the game (Yang, 2007). Presently, the majority of national and international referees originate from university sports faculties or other professionals within the sports sector, indicating a need in the cultivation of the basketball officiating workforce (Wang, 2015). This study employs college students enrolled in sports education programs as experimental subjects based on these findings. The trend of a younger referee workforce and heightened standards for personal proficiency confer distinct advantages to students from basic university sports education programs. National policies may facilitate focused working memory training for this group, perhaps improving their officiating decision-making skills during games and so fostering the healthy advancement of the national sports business.

In cognitive psychology, working memory generally denotes a limited-capacity system that temporarily maintains and processes information needed for complex cognition (Ripp et al., 2022). Recent evidence indicates that targeted working memory training can produce small improvements in working memory and closely related tasks, but transfer to fluid intelligence, inhibitory control, attention, judgement, and decision-making is inconsistent (Hocking et al., 2020; Ripp et al., 2022; Syed et al., 2024; You et al., 2021). Furthermore, sport research indicates that decision-making training can improve sports officials’ performance, although effects vary by sport, outcome, and training design (Kittel et al., 2025; Larkin et al., 2018). Working memory is one of the general cognitive functions studied in relation to sports expertise and decision-making, but evidence is stronger for sport-specific cognitive skills than for general executive-function tests (Furley et al., 2023; Kalén et al., 2021). Working memory is crucial in sports, especially for basketball officials. Research on collegiate basketball referees indicates that those possessing superior working memory capacity demonstrate enhanced decision-making accuracy (Gou and Li, 2023). Referees must consistently monitor the actions of numerous players while being vigilant to the rules and dynamics of the game, necessitating a substantial capacity for working memory and attention management. Recent eye-tracking research shows that expert basketball referees make more accurate decisions and allocate more gaze time to task-relevant areas than non-experts (Wang et al., 2025). Consequently, perceptual-cognitive assessment may inform the development of collegiate basketball referees and support officiating standards.

Collegiate basketball referees routinely render officiating decisions that conform to sports decision-making principles. Referees frequently make quick decisions under significant pressure from time limits, psychological stress, coaches, and fans to facilitate the smooth course of the game and assure timely decision-making. As basketball regulations progress, the expectations for referees have intensified. Referees must now ground their choices on the entirety of the action rather than the moment it transpires, so augmenting their demand for cognitive resources. From a training standpoint, it is crucial to investigate if working memory training can elevate referees’ working memory capacities and hence boost their decision-making skills.

In conclusion, studies on the decision-making of collegiate basketball referees from a cognitive psychology viewpoint are scarce, especially regarding the influence of working memory training on their judgements. National regulations endorse collegiate basketball referees as an emerging segment of the officiating industry; nonetheless, there is a paucity of studies examining the impact of working memory training on their decision-making capabilities. In this context, examining the impact of working memory training on collegiate basketball referees’ officiating choices is crucial for enhancing the quality of development within the referee community. In light of these data, the subsequent hypothesis are posited:

*H1:* Collegiate basketball referees who get working memory training will demonstrate a marked enhancement in decision-making accuracy relative to the untrained control group.

*H2:* Collegiate basketball referees who participate in working memory training will demonstrate enhanced decision-making speed relative to the untrained control group.

*H3:* Collegiate basketball referees who participate in working memory training will demonstrate a substantial improvement in working memory capacity relative to the control group that receives no training.

## 2 Subjects and methods

### 2.1 Participants

A total of 32 collegiate basketball officials were first selected from a sports education program at a university in Jiangsu Province, China. Before participation, all candidates went through systematic screening according to pre-defined inclusion and exclusion criteria. Four individuals were excluded during screening: two had chronic physical conditions that prevented completing the study, and two failed visual screening and met the exclusion criteria. Following screening, 28 qualified subjects were incorporated into the final analysis. All 28 randomized participants completed the full 8-week intervention and were included in the final data analysis; there were zero drop-outs in this trial. Participants were randomly allocated to the experimental group (*n* = 14; 10 males, 4 females) or the control group (*n* = 14; 12 males, 2 females). Sample size estimate was performed utilising G*Power 3.1, with the assumption of a medium effect size (*f* = 0.25), *α* = 0.05, and statistical power (1 − *β*) = 0.80. A minimum of 14 participants per group was necessary, accounting for a potential attrition rate of 20%.

### 2.2 Criteria for inclusion and exclusion

#### 2.2.1 Inclusion criteria

1. The referees must possess a minimum qualification of second-tier level or higher, have normal or corrected eyesight, and be free from conditions such as astigmatism or colour blindness. All participants must be dextral.
2. Academic background: Since the majority of collegiate basketball referees are from sports universities, the study focused on collegiate basketball referees enrolled in sports education programs.
3. Participants are required to possess a requisite level of basketball officiating experience, including prior service as referees in municipal or county-level events, so assuring a foundational comprehension of officiating judgements.
4. Language understanding and expression: Participants must demonstrate the capacity to comprehend and articulate language clearly, as referees are required to interact effectively with players and fellow refs.

#### 2.2.2 Exclusion criteria

1. Having chronic physical illnesses that prevent completing the full 8-week training and testing procedure;
2. Visual defects including astigmatism, colour blindness, or colour weakness that cannot meet the visual requirements of experimental tasks;
3. Being unable to fully understand experimental instructions and finish all experimental tasks;
4. Being unable to guarantee attendance for all training sessions, pre-test and post-test assessments.

### 2.3 Experimental controls

1. Uniformity of referee qualifications and age categories between the experimental and control groups.
2. Fixed duration and timing of working-memory training sessions were applied for the experimental group; the blank control group received no cognitive training intervention.
3. Control of human factors: Demographic characteristics including gender and age were collected via E-Prime. Only gender was entered as a covariate in statistical models; age was not included in the analysis.
4. The experiment was performed in a tranquil, undisturbed setting to guarantee little interference.

### 2.4 Experimental design

*Procedure 1:* Assess the working memory capacities of both the experimental and control groups prior to and subsequent to the experiment.

*Procedure 2:* A mixed-factorial experimental design was employed, consisting of 2 (experimental group, control group) × 2 (pre-test, post-test) × 2 (cognitive decision-making, intuitive decision-making). The referee groups (experimental and control) constituted the between-subjects variable, whilst time and decision task type represented the within-subjects factors. The dependent variables were reaction time and accuracy for both intuitive and cognitive decision-making tasks. The decision task was categorised into cognitive and intuitive decision-making tasks.

The experimental group participated in an 8-week intervention consisting of three uninterrupted training sessions of 30–35 min each week, whereas the control group received no training but was otherwise treated identically to the experimental group. In the current study, the control group received no cognitive intervention (blank control). An active placebo-training control condition was not implemented, which represents a limitation of the present design.

### 2.5 Experimental procedures

#### 2.5.1 Working memory training programs

1. Stroop task (training inhibitory function) (see Figures 1, 2).
2. 2-back task (training updating function) (see Figures 3, 4).
3. More-odd shifting task (training shifting function) (see Figures 5, 6).

**Figure 1:**
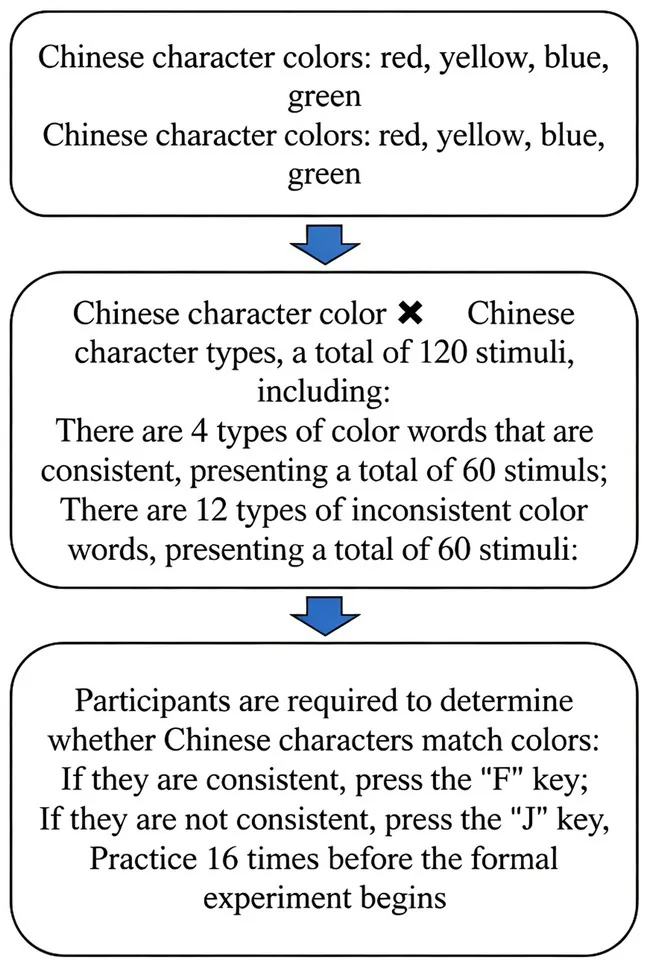
Schematic flowchart of the Stroop task for inhibitory‑control training, illustrating stimulus types (colour‑consistent and colour‑inconsistent Chinese character stimuli), total number of trials, and participant response instructions.

**Figure 2:**
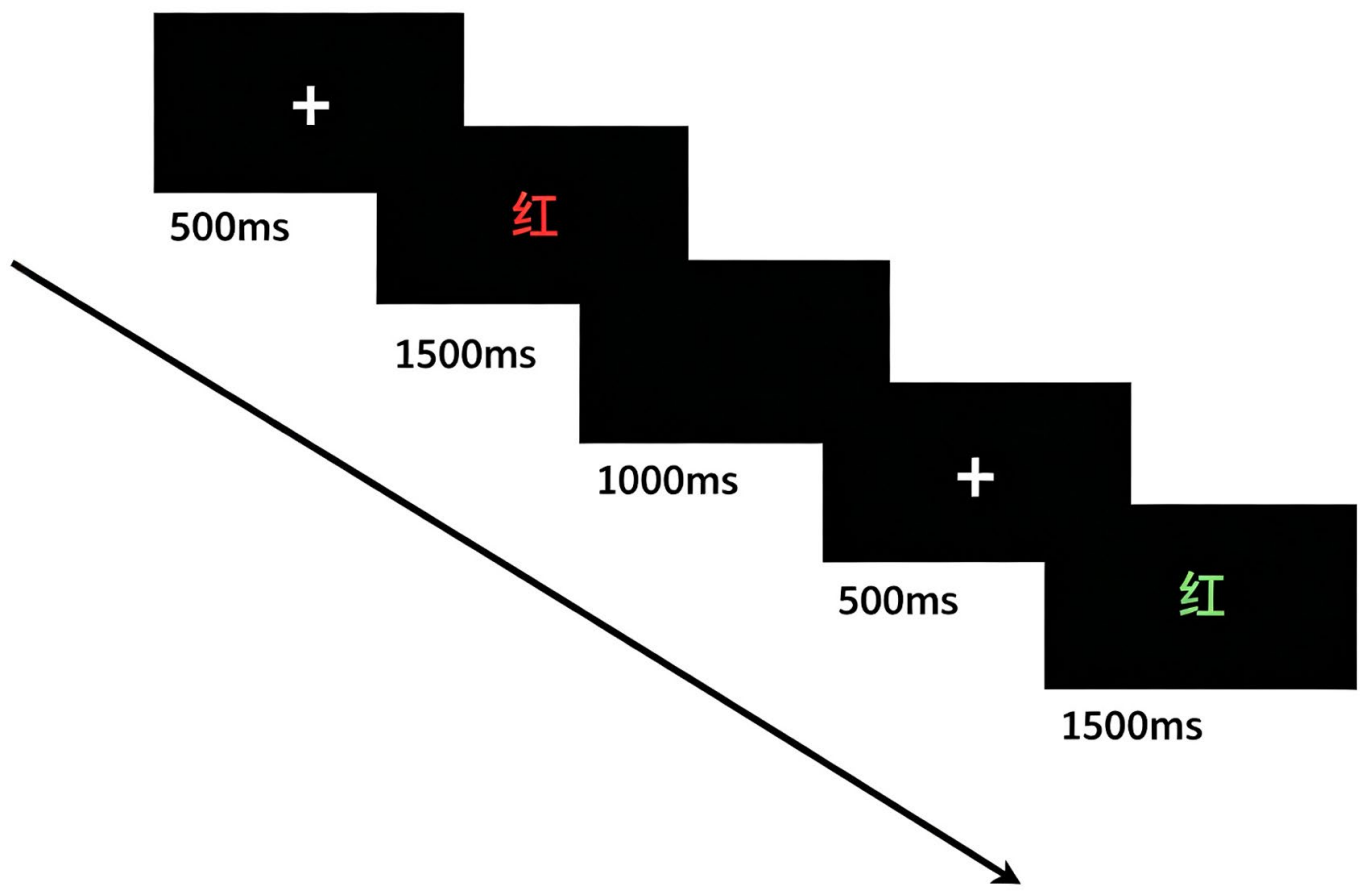
Single‑trial time‑course diagram of the Stroop task, showing the sequence and duration of fixation cross, character stimulus presentation, and inter‑stimulus interval.

**Figure 3:**
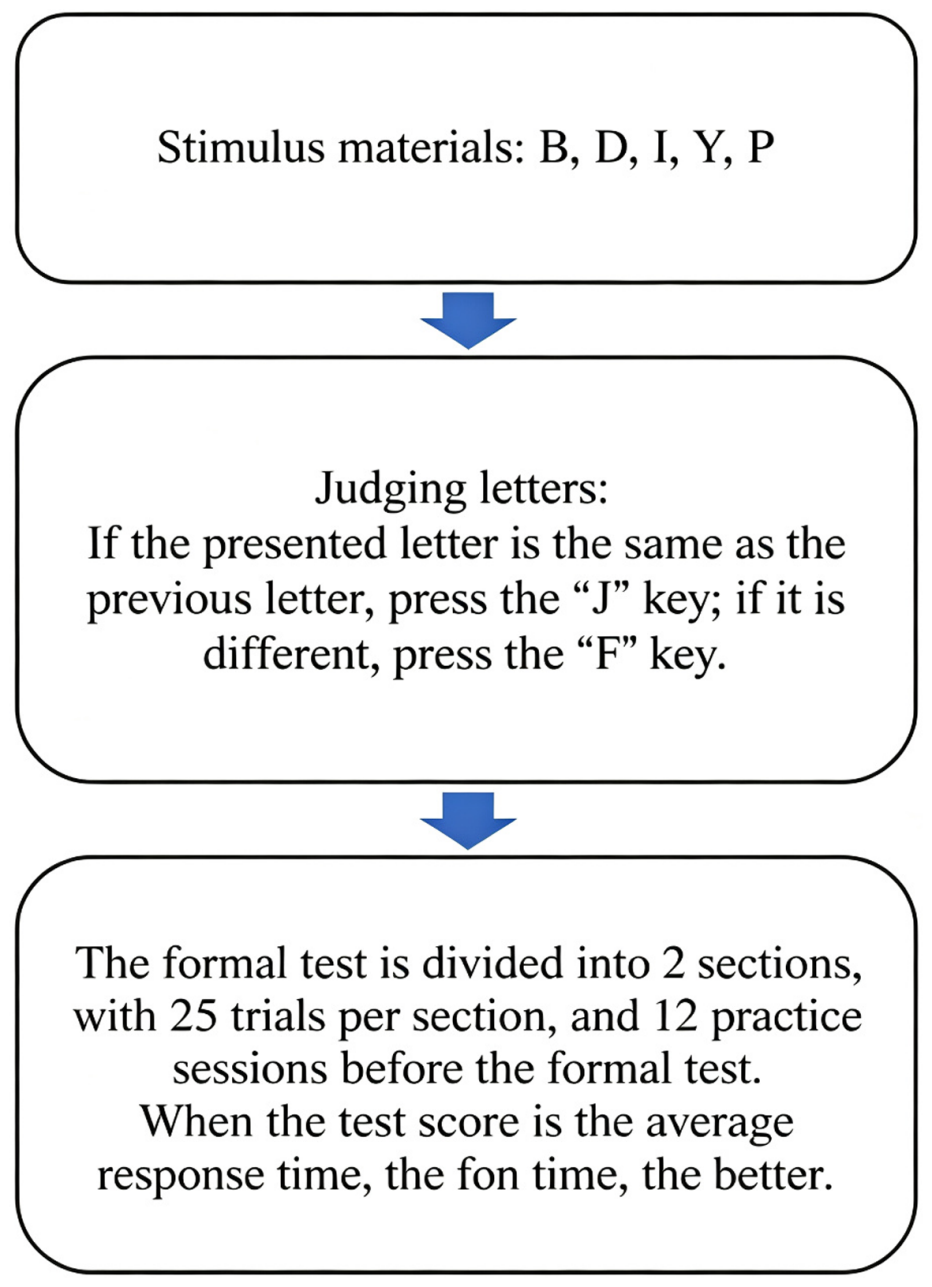
Schematic of the 2‑back working‑memory updating task, including stimulus materials (letters B, D, I, Y, P), response rules, formal‑test block arrangement and practice trials.

**Figure 4:**
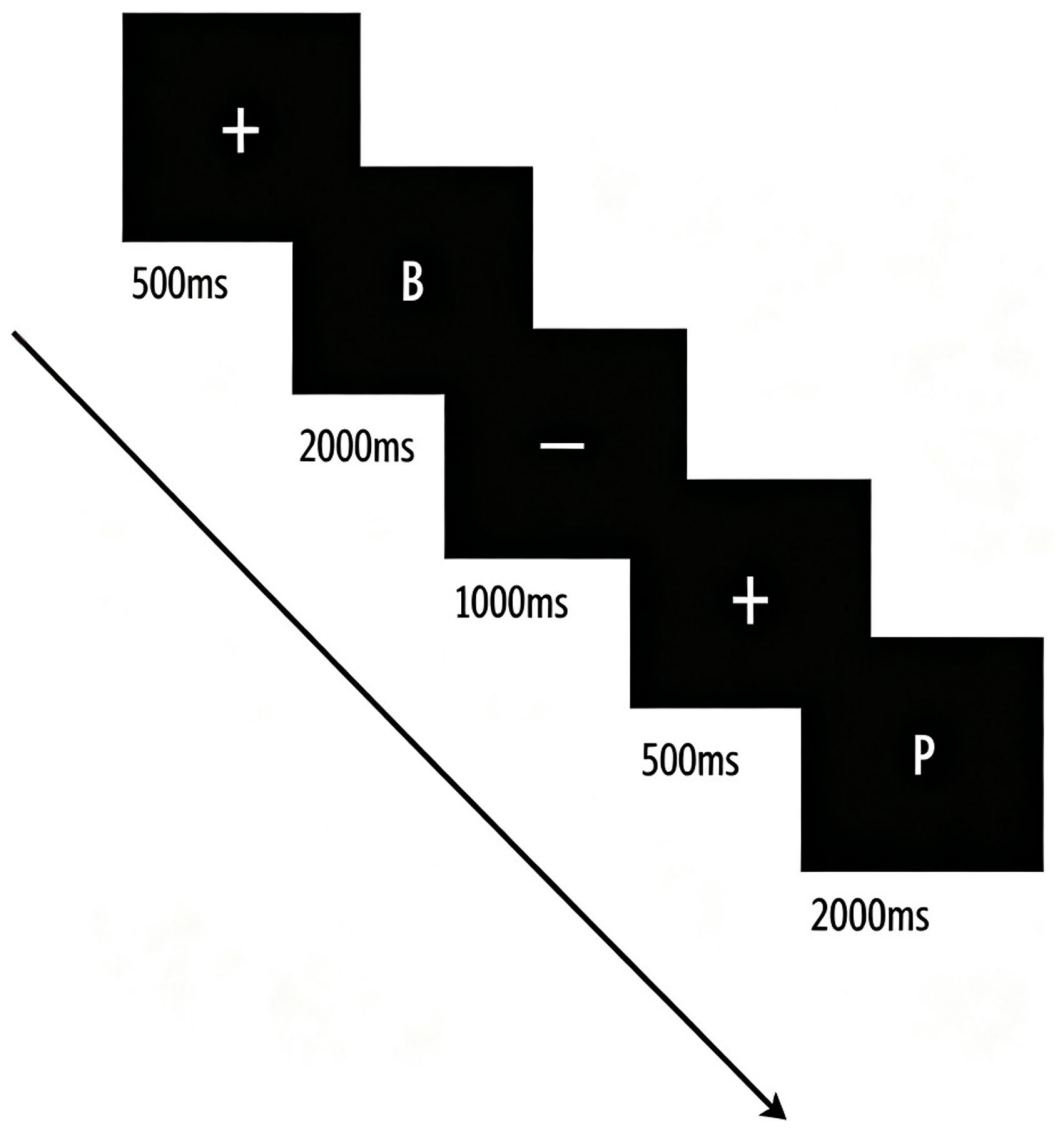
Timeline workflow for one complete trial within the 2‑back task, displaying the temporal sequence of fixation cues and letter stimuli with corresponding millisecond durations.

**Figure 5:**
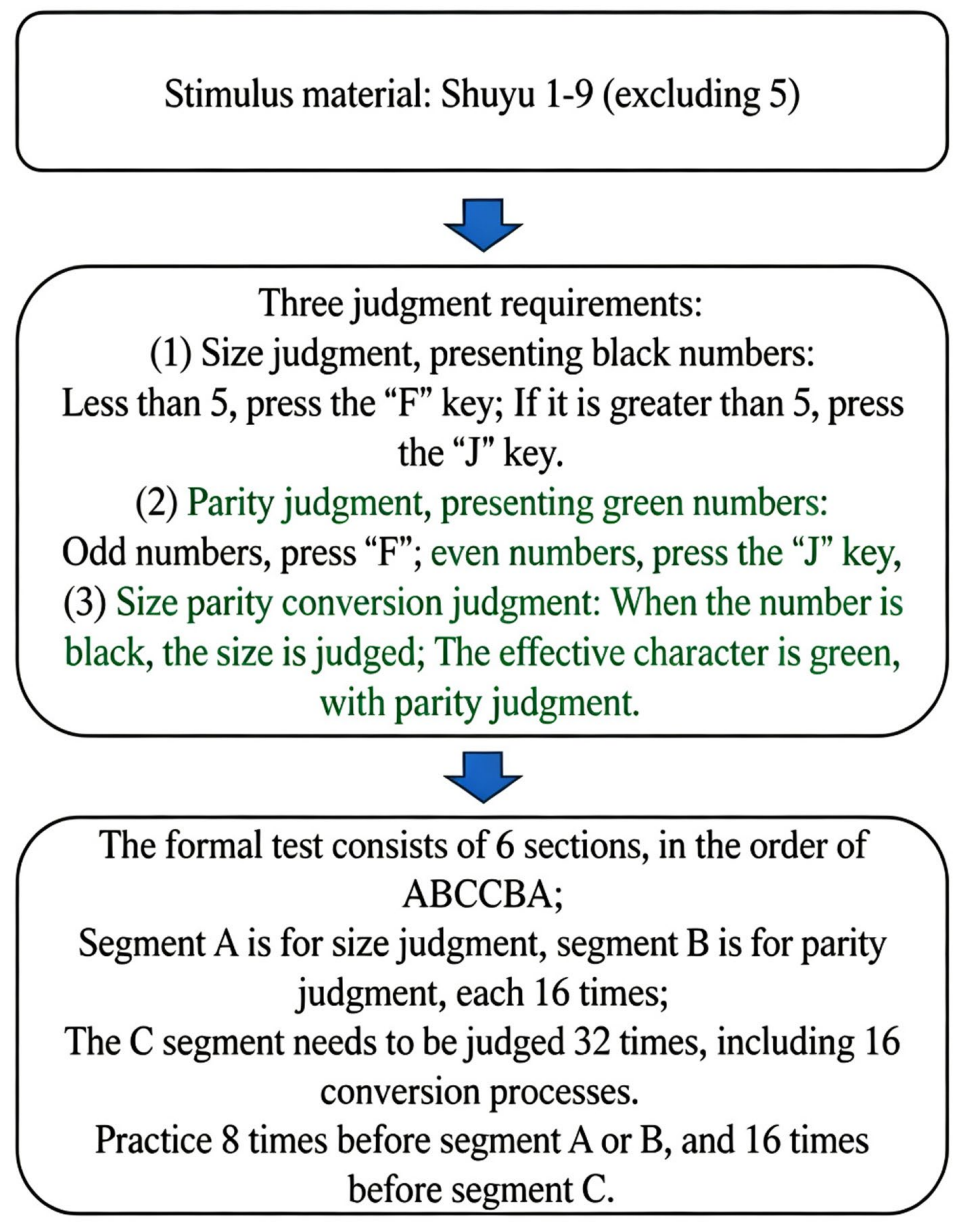
Flowchart of the More‑Odd Shifting task for mental‑set shifting training, covering stimulus range (digits 1–9 excluding 5), three judgment types (size judgment, parity judgment, size‑parity switching) and test block sequence.

**Figure 6:**
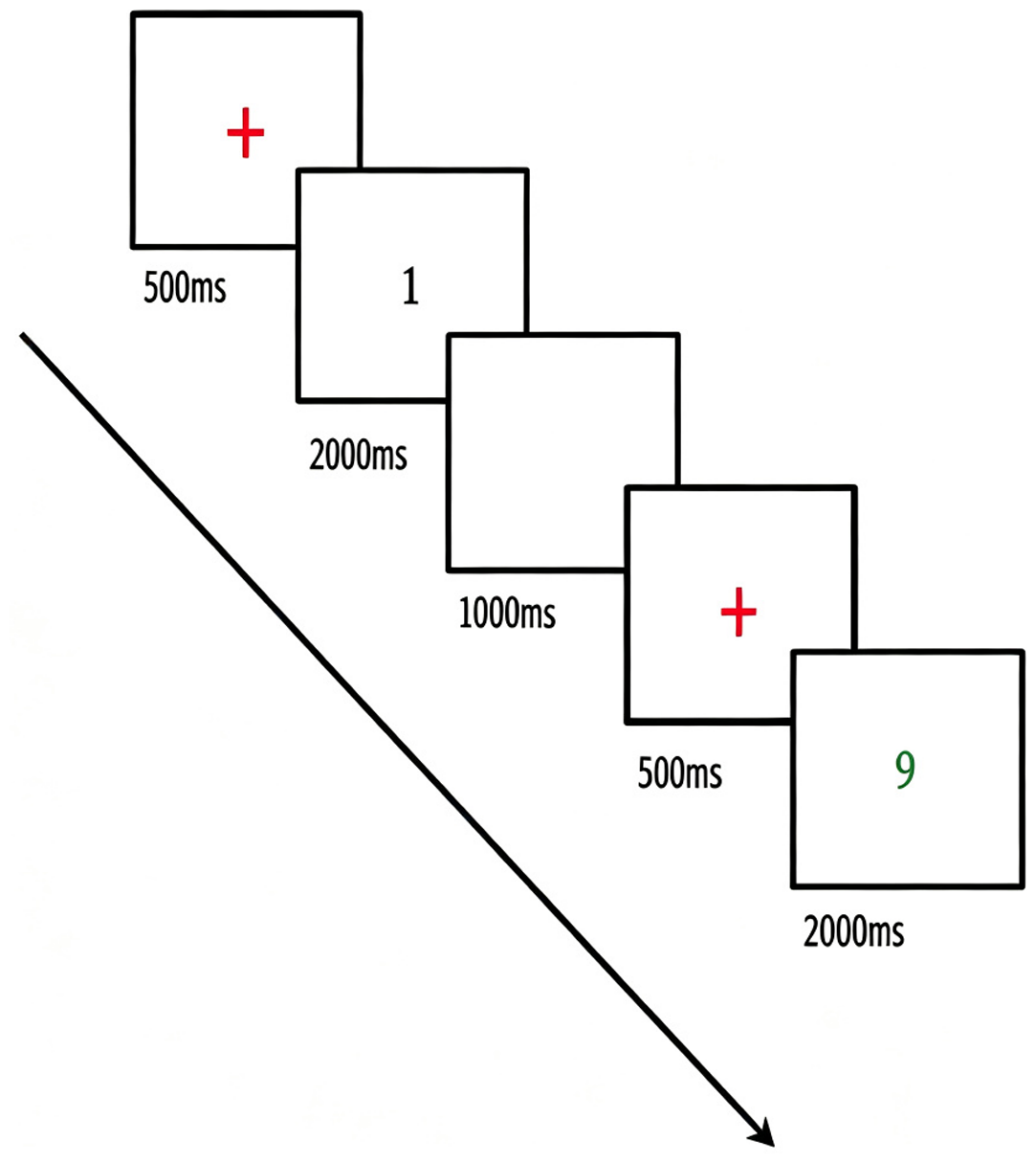
Time‑course diagram for a representative trial from the More‑Odd Shifting task, presenting fixation markers, digit stimuli and time parameters across task progression.

#### 2.5.2 Working memory span task

The working memory span assignment was created on E-prime 2.0 alongside WPS Office software. The working memory capacity of 28 collegiate basketball referees was assessed using a complex-span paradigm (Richmond et al., 2022). A total of 67 lines from the 2022 FIBA basketball regulations were utilised, each accompanied by a randomly chosen uppercase letter. The complexity and suitability of sentences were assessed by reviewers of varying expertise to assure validity. The complexity of the tasks escalated incrementally, comprising as many as six sentences per set. Participants received one point for each accurately recalled letter, with a maximum score of 120. Processing capacity was indicated by the accuracy of rule judgements, and storing capacity was assessed through letter recall. The operational span score was determined by summing the processing and storage scores and dividing by the total number of trials (Ni, 2017) (see Figure 7).

**Figure 7:**
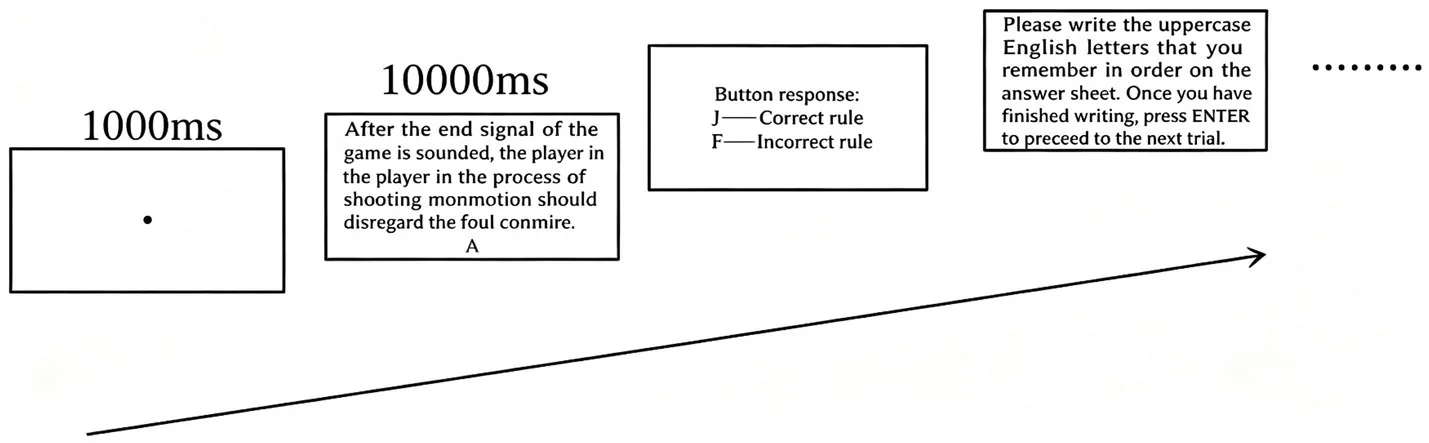
Schematic diagram of the complex‑span working‑memory span task built in E‑Prime 2.0, using sentences excerpted from official FIBA basketball regulations together with random uppercase‑letter recall requirements.

#### 2.5.3 Image selection for officiating decision-making tasks

High-definition photos from NBA and CBA highlights, measuring 1,436 × 1988 pixels, were processed using Photoshop. Initially, 58 photos of cognitive decision-making and 62 images of intuitive decision-making were chosen, comprising equal quantities of foul and non-foul scenarios. Images were evaluated by referees of varying expertise to eliminate ambiguous or contentious cases and subsequently validated by a test group. According to previous studies, the final stimuli exhibited a decision accuracy range of 30 to 80% (Cheng, 2006; Wang et al., 2022; Ye, 2012). In the structured experiment, participants first completed 50 formal intuitive-decision-making images presented for 400 ms (25 foul, 25 non-foul), followed by 50 formal cognitive-decision-making images presented for 1,000 ms (25 foul, 25 non-foul). Six separate images were used for pre-experimental practice. No block-order counterbalancing was implemented in this study. Presentation durations were established based on prior research (Feng et al., 2019; Wang, 2004; Wang et al., 2022; Ye, 2012). Six separate images were used only for pre-experimental practice and were not part of these 50 formal trials.

#### 2.5.4 Officiating decision-making procedure

The officiating decision-making process was created utilising E-Prime 2.0 software and has two modules: Module 1 focuses on intuitive decision-making, whereas Module 2 addresses cognitive decision-making. Participants first undertake six independent practice trials before the commencement of the formal experiment; these practice trials are not counted within the 50 formal trials per decision-making condition. The method commences with the display of a fixation point for 1,000 ms, succeeded by the presentation of a stimulus image for 400 ms (for cognitive decision-making, the image is displayed for 1,000 ms). Participants must determine if a foul is present in the image and answer by hitting a key: “J” for no foul and “F” for a foul. A blank screen is presented for 3,000 milliseconds. If no key is pressed during this duration, the trial automatically advances to the subsequent one. Upon concluding the practice for Module 1, a 3-min interval is provided to maintain participants’ concentration on the ongoing experiment (pictures are displayed randomly) (see Figure 8).

**Figure 8:**
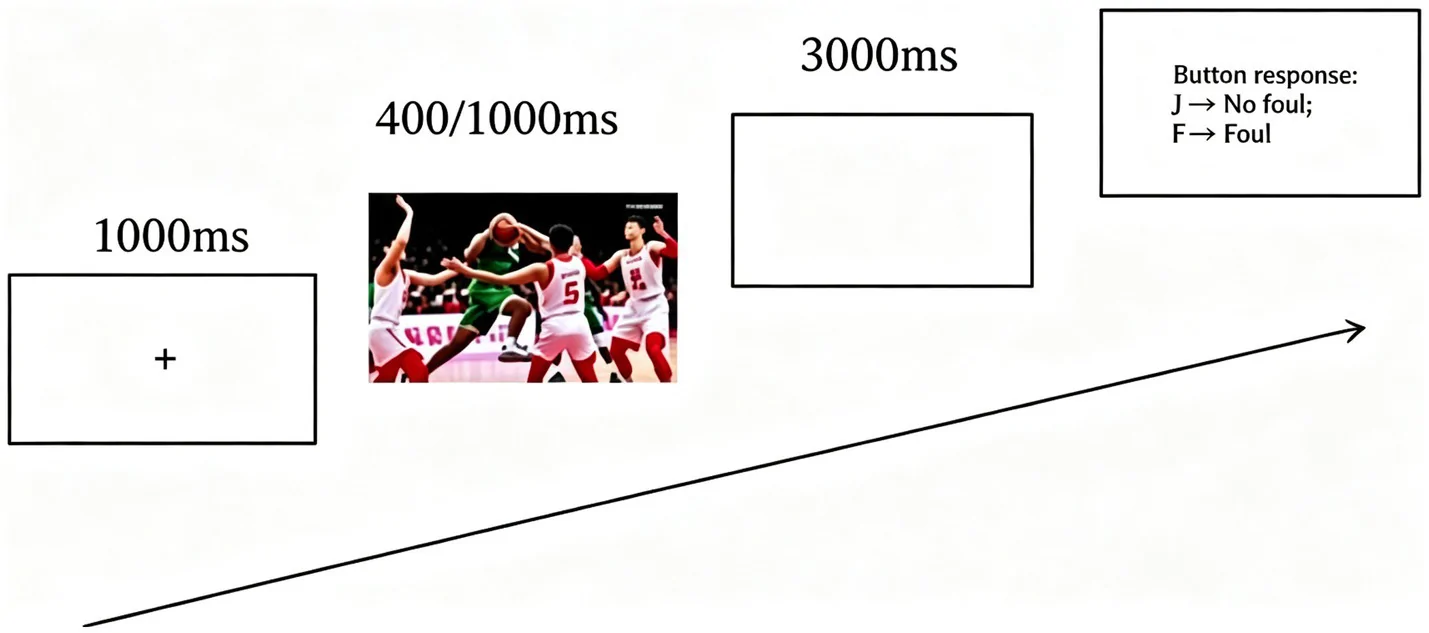
Experimental timeline of the basketball‑referee officiating‑decision paradigm: 1000 ms fixation cross, 400 ms (intuitive decision) / 1000 ms (cognitive decision) stimulus image presentation, 3000 ms blank response window; participants press “J” for no‑foul and “F” for foul judgements.

### 2.6 Experimental procedure

The 28 volunteers were assembled, briefed on the experimental process, and requested to sign the informed consent form. Every three individuals were allocated to groups and subsequently headed to the laboratory to undertake the working memory span test. Following a 10-min interval, participants undertook the officiating decision-making assessment, which included a 3-min interval separating the intuitive decision-making group from the cognitive decision-making group. Participants were acknowledged for their involvement and presented with little tokens of appreciation. Prior to group allocation, participants were stratified according to their referee level. Within each stratum, a random-number table was used to assign participants to either the experimental group or the control group. The experimental group participated in 8 weeks of working memory training, consisting of three training sessions of 30–35 min each week, incorporating the Stroop test, 2-back task, and More-Odd shifting problem. Following the 8-week working memory training, post-tests were administered to assess working memory capacity, decision-making accuracy, and response time for both the experimental and control groups.

### 2.7 Statistical analysis

All statistical analyses were conducted using SPSS 26.0 for Windows, with the statistical significance level set at 0.05.

1. Baseline comparisons: independent-samples t-tests were used to compare pre-test officiating accuracy, reaction time, and working-memory-capacity scores between the experimental and control groups for both intuitive and cognitive decision-making conditions.
2. Within-group pre-post changes: paired-samples *t*-tests were applied to test within-group changes in officiating accuracy, reaction time and working-memory capacity before and after intervention for the experimental group and the control group separately.
3. Primary outcome models (officiating accuracy and reaction time): a 2 (group: experimental/control, between-subject factor) × 2 (time: pre-test/post-test, within-subject factor) × 2 (decision-making type: intuitive/cognitive, within-subject factor) mixed-factorial repeated-measures analysis of variance (ANOVA) was performed. Gender was entered into each model as a covariate.
4. Follow-up for significant interactions: simple-effect analyses were conducted to decompose statistically significant interaction effects. No multiple-comparison adjustment was applied for simple-effect follow-up tests, and this limitation is explicitly noted. No additional pairwise comparisons were carried out.
5. Effect-size reporting: partial eta-squared (*ηp*^2^) was reported as the effect-size metric for repeated-measures ANOVA; Cohen’s *d* was reported for t-test outcomes.
6. Reaction-time pre-processing: prior to reaction-time statistical analysis, error trials and non-response trials were excluded. Outlying reaction-time values beyond ±3 standard deviations from each participant’s individual mean were also removed.

All analytical procedures strictly follow the original study workflow, and no original statistical outputs were modified.

## 3 Results

### 3.1 The impact of working memory training on decision-making accuracy in collegiate basketball officiating

#### 3.1.1 Homogeneity test of officiating decision accuracy between the experimental and control groups prior to the experiment

An independent samples *t*-test was conducted to compare the pre-test decision accuracy between the experimental and control groups for both intuitive and cognitive decision types. As presented in Table 1, no significant differences were found between the two groups for either decision type, indicating no statistically significant baseline difference was detected between the two groups.

**Table 1:** Independent samples *t*-test of pre-test decision accuracy by group and decision type.

Accuracy	Experimental group (*N* = 14)	Control group (*N* = 14)	*t*	*p*
Intuitive	0.703 ± 0.060	0.700 ± 0.044	0.144	0.887
Cognitive	0.781 ± 0.064	0.764 ± 0.049	0.792	0.436

#### 3.1.2 Repeated measures ANOVA results for decision accuracy with gender as a covariate

A repeated measures ANOVA was performed with decision accuracy as the dependent variable, and time (pre-test, post-test), group (experimental, control), and decision type (intuitive, cognitive) as independent variables, with gender included as a covariate. The results are summarized in Table 2.

**Table 2:** Repeated measures ANOVA of decision accuracy by time, group, and decision type with gender as covariate.

Effect	Type III SS	df	Mean square	*F*	*p*	*η^2^_p_*
Time	0.016	1	0.016	4.488	0.044*	0.152
Group	0.074	1	0.074	16.381	*p* < 0.001**	0.396
Decision Type	0.01	1	0.01	5.825	0.023*	0.189
Time × group	0.048	1	0.048	13.749	0.001**	0.355
Time × decision type	0	1	0	0.026	0.872	0.001
Group × decision type	0.001	1	0.001	0.554	0.464	0.022
Time × group× decision type	0.000	1	0.000	0.022	0.882	0.001
Gender	0.002	1	0.002	0.51	0.482	0.02
Time × gender	0.008	1	0.008	2.191	0.151	0.081
Decision type ×gender	0.000	1	0.000	0.252	0.62	0.01
Time × decision type × gender	0.000	1	0.000	0.061	0.807	0.002

**p < 0.05; *p < 0.01.

The analysis revealed significant main effects for time, group, and decision type. *Post-hoc* comparisons indicated that decision accuracy was significantly higher in the post-test than in the pre-test, and that the experimental group significantly outperformed the control group overall. Additionally, cognitive decisions were made with significantly greater accuracy than intuitive decisions. A significant interaction was found between time and group. Simple effects analysis (Figure 9) showed that while no group difference existed at pre-test, the experimental group demonstrated significantly higher decision accuracy than the control group at post-test. No other interactions reached significance, and the main effect of gender, as well as its interactions with other factors, were not significant. These findings indicate that the 8-week working memory intervention effectively enhanced the decision-making accuracy of collegiate basketball referees.

**Figure 9:**
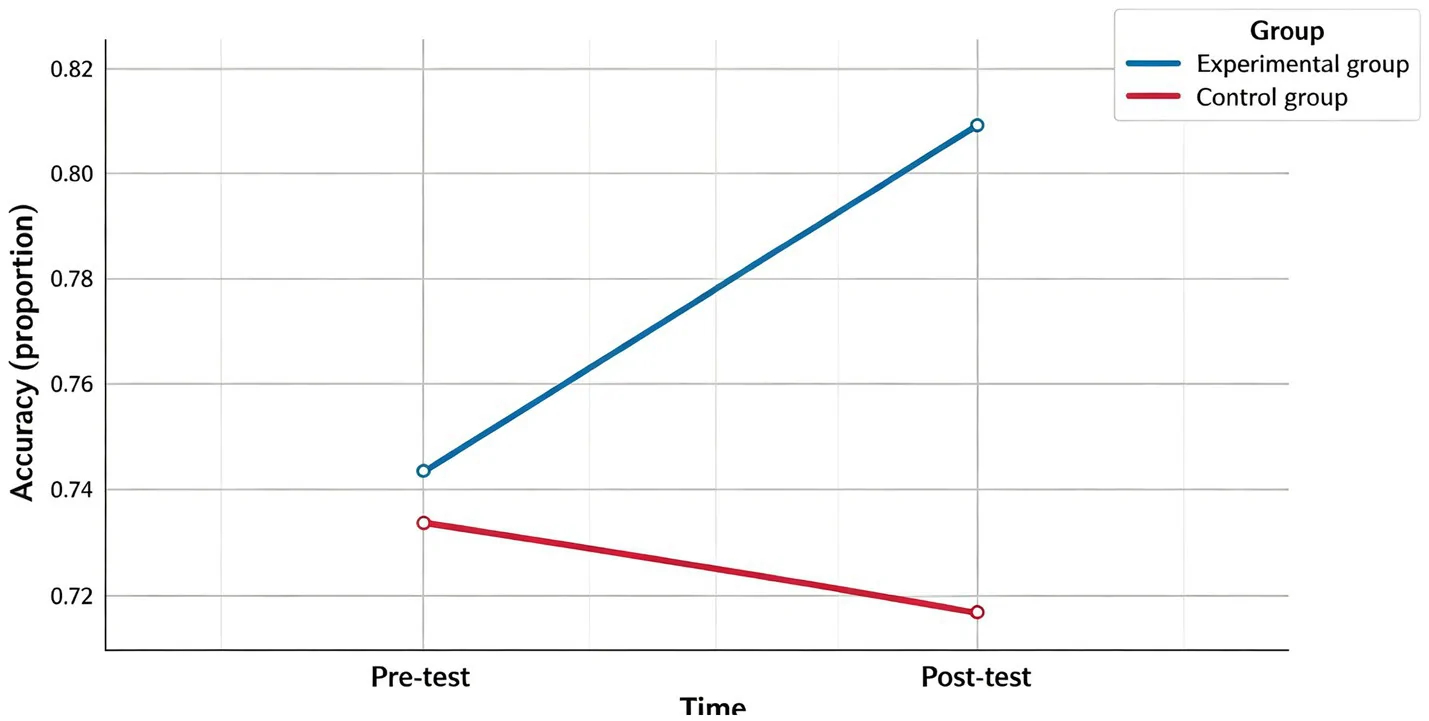
Changes in decision accuracy before and after the intervention in collegiate basketball referees across different groups.

### 3.2 The effect of working memory training on officiating decision reaction time in collegiate basketball referees

#### 3.2.1 Homogeneity test of officiating decision reaction time between the experimental and control groups before the experiment

An independent samples t-test on pre-test reaction times (Table 3) revealed no significant differences between the experimental and control groups for either intuitive or cognitive decision-making, showing no statistically significant baseline difference detected between groups.

**Table 3:** Independent samples *t*-test of pre-test decision reaction time by group and decision type.

Reaction time	Experimental group (*N* = 14)	Control group (*N* = 14)	*t*	*p*
Intuitive dec.	1158.72 ± 90.78	1146.31 ± 126.95	0.298	0.768
Cognitive dec.	1028.99 ± 83.08	1051.19 ± 101.93	−0.632	0.533

#### 3.2.2 Repeated measures ANOVA results for decision reaction time with gender as a covariate

A repeated measures ANOVA, with gender as a covariate, was conducted on decision reaction time. The results are presented in Table 4. Significant main effects were found for time and group, indicating that reaction times were significantly shorter in the post-test than in the pre-test, and that the experimental group responded significantly faster overall than the control group. The main effect of decision type was not significant, although a consistent trend for faster cognitive responses was observed across time points (Figure 10).

**Table 4:** Repeated measures ANOVA of decision reaction time by time, group, and decision type with gender as covariate.

Effect	Type III SS	df	Mean Square	*F*	*p*	*η^2^_p_*
Time	43451.844	1	43451.844	5.848	0.023*	0.19
Group	222735.971	1	222735.971	17.310	*p* < 0.001**	0.409
Decision type	16004.444	1	16004.444	1.274	0.27	0.048
Time × group	198953.059	1	198953.059	26.778	p < 0.001**	0.517
Time × decision type	33576.72	1	33576.72	3.151	0.088	0.112
Group × decision type	26659.337	1	26659.337	2.122	0.158	0.078
Time× group × decision type	64947.613	1	64947.613	6.095	0.021*	0.196
Gender	2758.178	1	2758.178	0.214	0.647	0.009
Time × gender	8268.368	1	8268.368	1.113	0.302	0.043
Decision type × gender	608.571	1	608.571	0.048	0.828	0.002
Time × decision type × gender	22682.603	1	22682.603	2.129	0.157	0.078

^*^p < 0.05; ^**^p < 0.01.

**Figure 10:**
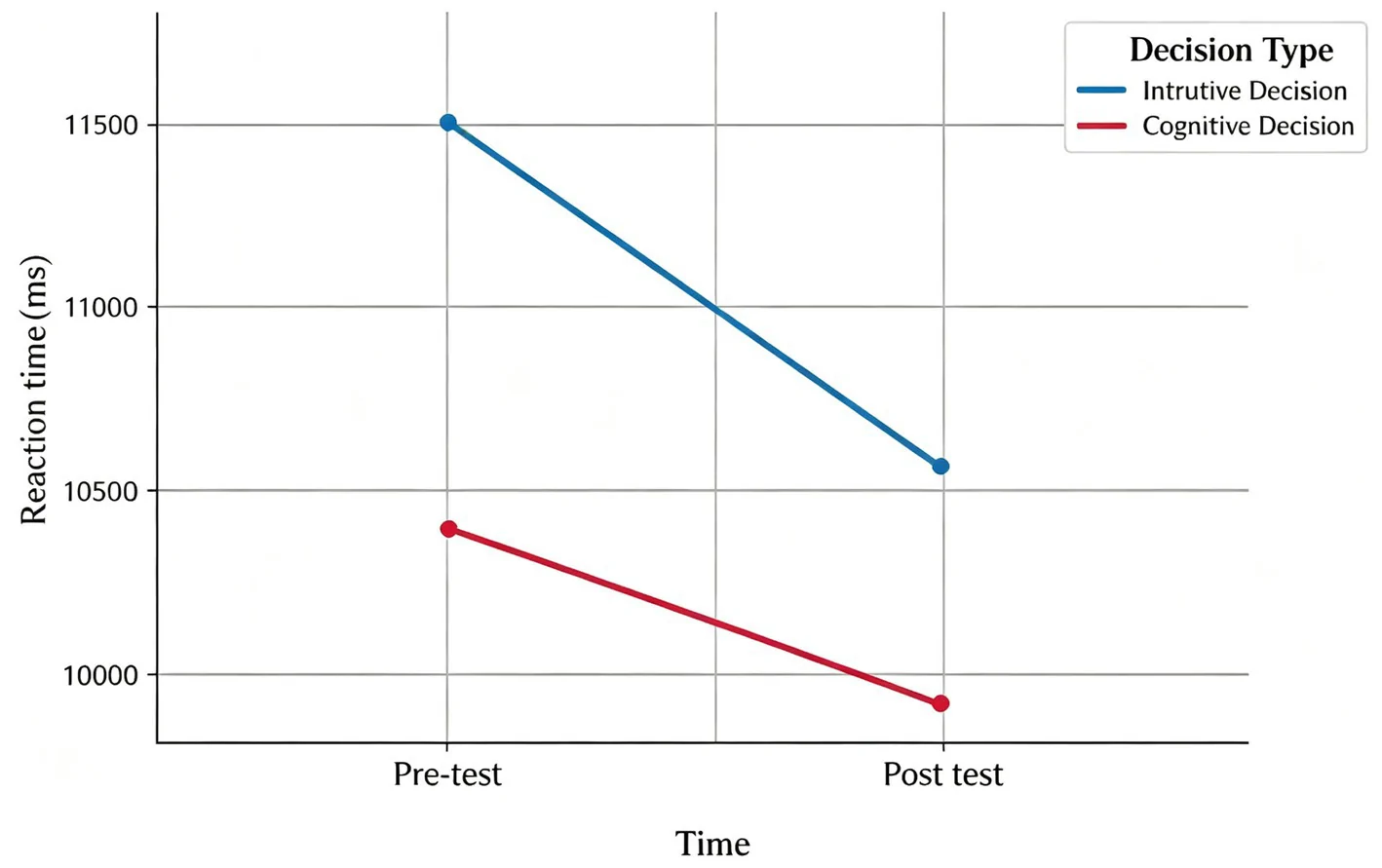
Trends in decision reaction time before and after the experiment under different decision types.

A significant time × group interaction emerged. Simple effects analysis (Figure 11) showed that while no group difference existed at pre-test, the experimental group exhibited significantly shorter reaction times than the control group at post-test.

**Figure 11:**
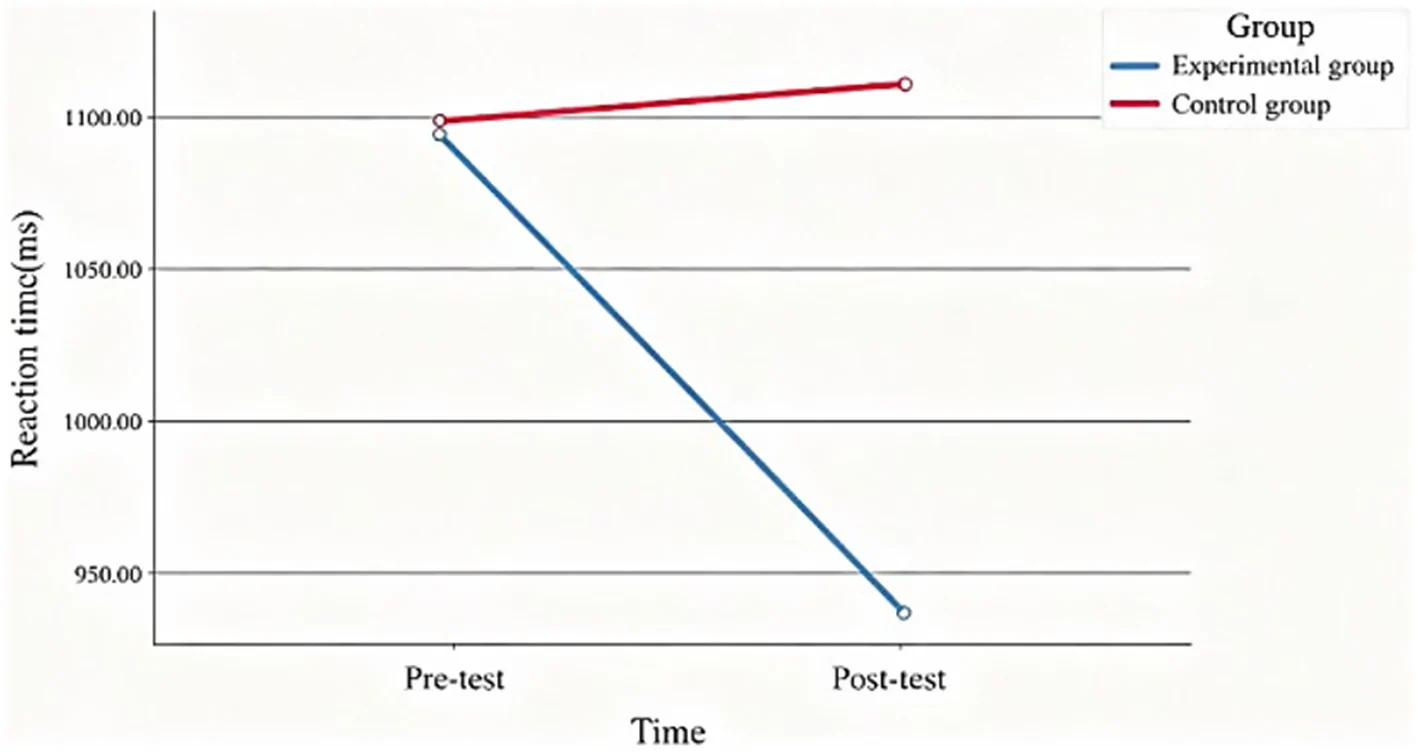
Time × group interaction for decision reaction time.

Furthermore, the three-way interaction among time, group, and decision type was significant. To decompose this interaction, separate simple effects analyses were performed for each decision type. In the intuitive decision condition, the experimental group showed a significant reduction in reaction time from pre- to post-test and significantly outperformed the control group at post-test (Figure 12). Similarly, in the cognitive decision condition, the same pattern of significant improvement and superior post-test performance was observed for the experimental group (Figure 13). No significant effects involving gender were detected. Collectively, these findings demonstrate that the training intervention significantly accelerated decision-making speed, with the benefit being particularly robust in the cognitive decision condition.

**Figure 12:**
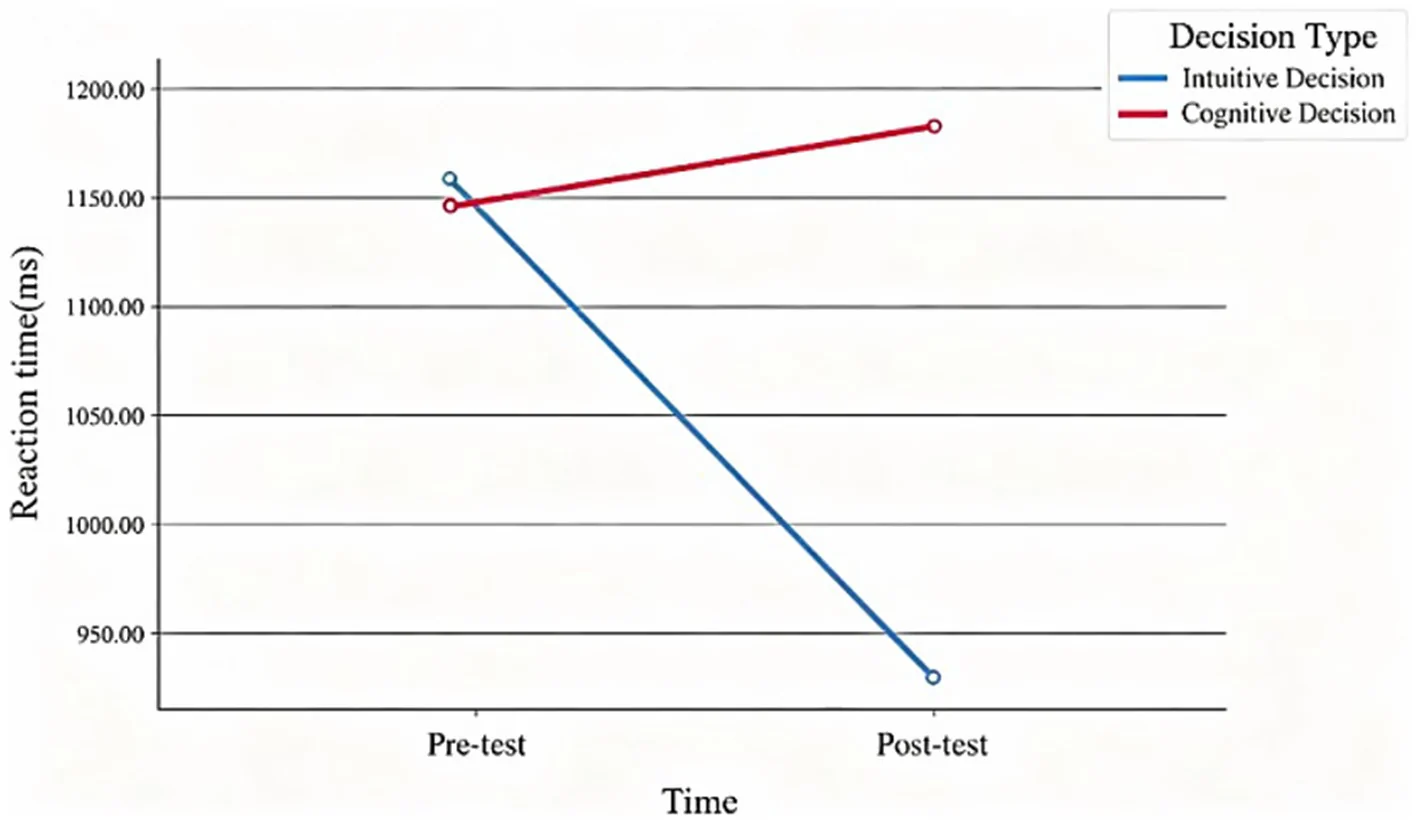
Changes in decision reaction time under intuitive decision-making across groups.

**Figure 13:**
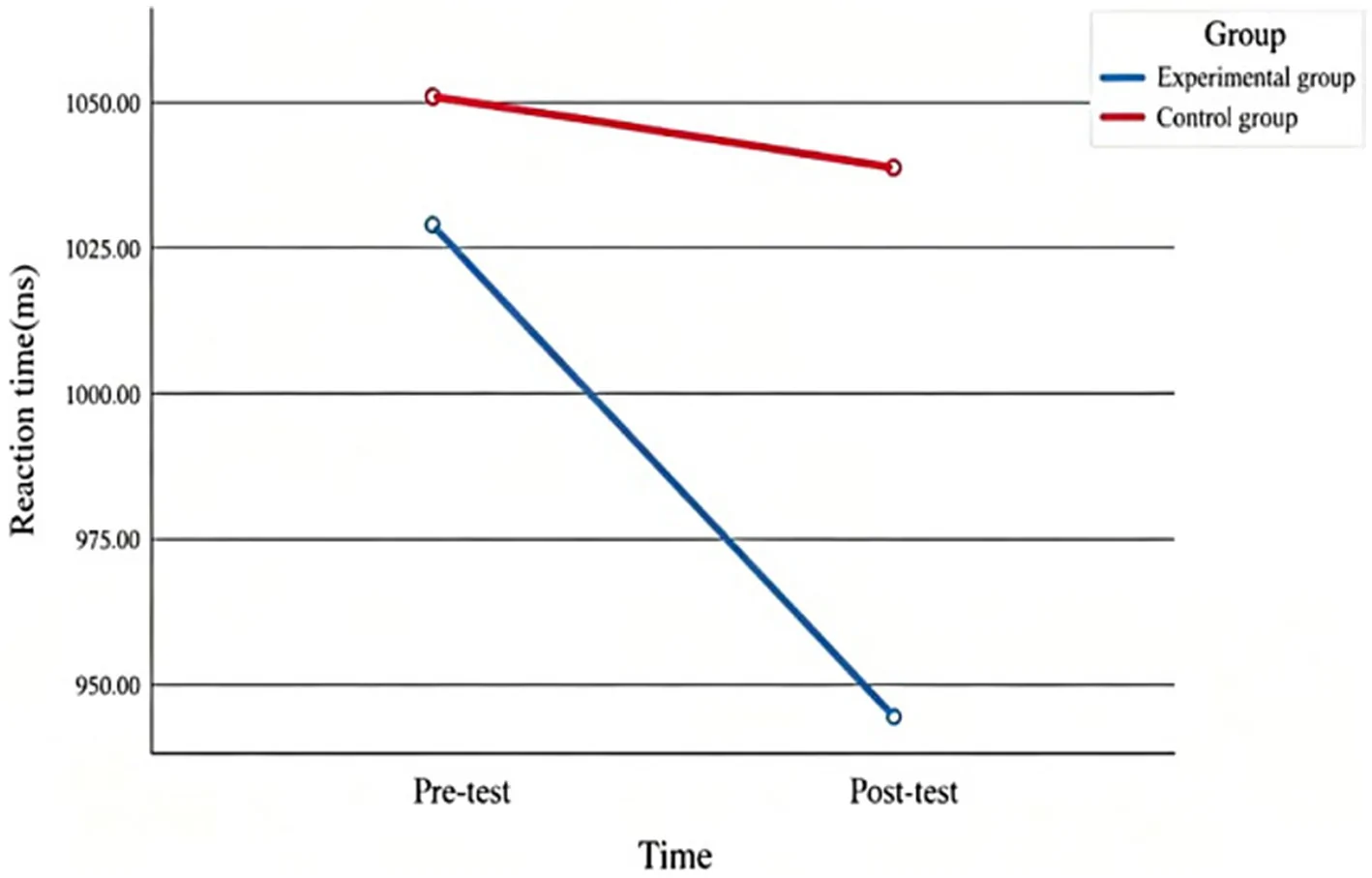
Changes in decision reaction time under cognitive decision-making across groups.

### 3.3 The effect of working memory training on working memory capacity in collegiate basketball referees

#### 3.3.1 Homogeneity test of working memory capacity between the experimental and control groups before the experiment

An independent samples *t*-test on pre-test working memory capacity scores (Table 5) revealed no significant difference between the experimental and control groups, no statistically significant baseline difference was detected between groups.

**Table 5:** Independent samples *t*-test of pre-test working memory capacity scores by group.

Working memory capacity	M	SD	df	*t*	*p*	*F*
Experimental group	91.14	6.237	26	−0.233	0.817	0.029
Control group	91.71	6.707				

#### 3.3.2 Trends in changes of working memory capacity scores before and after the experiment in collegiate basketball referees

Paired samples *t*-tests were used to examine within-group changes from pre- to post-test (Table 6). The experimental group demonstrated a statistically significant and substantial increase in working memory capacity scores following the 8-week intervention. In contrast, no significant change was observed in the control group over the same period. These results indicate that the working memory training effectively enhanced participants’ working memory capacity.

**Table 6:** Paired samples *t*-test of working memory capacity scores by time.

Outcome measure	Group	*T*_1_	*T*_2_	*t*	(95% CI)	*p*
		Mean ±SD	Mean ±SD			
Verbal WM capacity^a^	IG	91.143 ± 6.237	96.500 ± 6.723	−7.625	−6.870∽ − 3.845	*p* < 0.001^**^
	CG	91.714 ± 6.707	92.000 ± 6.361	−0.269	−2.578∽2.006	0.792

IG, experimental group; CG, control group; [95% CI], 95% confidence interval. *T*_1_ indicates baseline assessment (first assessment), *T*_2_ indicates assessment at 8 weeks (second assessment).

a Paired samples *t*-test used to compare within-group changes from *T*_1_ to *T*_2_.

*p < 0.05; **p < 0.01.

### 3.4 The effect of working memory training on officiating decision accuracy in collegiate basketball referees

This study conducted an 8-week working memory training program for collegiate basketball referees to examine its impact on the correctness of refereeing decisions. The outcomes of homogeneity testing and repeated measures analysis revealed that post-working memory training, the experimental group exhibited markedly superior choice accuracy relative to the control group (*p* < 0.01), yielding medium-sized effect sizes. Importantly, these effects are limited to collegiate basketball referees with limited officiating experience and cannot be extrapolated to elite professional referee populations. Both temporal factors and group dynamics were determined to significantly affect choice accuracy. The accuracy in the post-test phase was markedly superior than that in the pre-test. Recent evidence indicates that decision-making training can improve performance in sports officials, with moderate overall effects but variation by sport, outcome type, and training design; basketball-referee research also links expertise with more accurate decisions and task-relevant visual search (Kittel et al., 2025; Wang et al., 2025). Together, this evidence supports a role for perceptual-cognitive training and visual attention in referee development, but it does not by itself establish that generic working memory training transfers to match decisions. The present study similarly demonstrated that cognitive decision-making accuracy regularly surpassed intuitive decision-making accuracy, both prior to and following the trial. This may be attributed to the following factors: cognitive decision-making typically entails more thorough and extensive information processing, wherein referees evaluate and interpret multiple data points prior to reaching a conclusion, potentially proving more effective than depending exclusively on intuition or experience. Recent research have shown the superiority of cognitive decision-making in referees’ judgements (Mann et al., 2019). The research indicated that experienced referees interpret visual information more efficiently and make more accurate decisions, consistent with recent basketball-referee eye-tracking evidence (Bergmann et al., 2021; Wang et al., 2025). A recent meta-analysis found an overall moderate effect of decision-making training in team-sport officials, while showing that effectiveness varies by sport and outcome type (Kittel et al., 2025). These findings collectively underscore the distinct benefits of cognitive decision-making in enhancing decision accuracy and consistency, particularly when referees must navigate intricate, rapidly evolving scenarios. Systematic cognitive training enables referees to process information more efficiently under pressure, make precise decisions, and enhance overall officiating standards.

Gender was used as a covariate to mitigate any confounding effects, and no significant impact on decision accuracy was seen. The findings suggest that working memory training can significantly improve decision accuracy among collegiate basketball referees with limited officiating experience, resulting in more dependable judgements through cognitive decision-making within laboratory static-image testing. Enhancing cognitive capacity and attentional control may assist referees in making more precise decisions during high-pressure, rapidly evolving laboratory simulated officiating tasks based on static images, thus fostering equitable and consistent officiating.

### 3.5 The effect of working memory training on officiating decision reaction time in collegiate basketball referees

The research utilised Stroop, 2-back, and More-Odd shifting tasks to enhance the central executive functions of working memory, specifically inhibition, updating, and shifting. Following 8 weeks of instruction, collegiate basketball referees in the experimental group exhibited markedly reduced decision reaction times in comparison to the control group. This finding is broadly consistent with meta-analytic evidence that computerized working memory training produces gains mainly on trained or closely related tasks, with smaller gains on untrained tasks (Li et al., 2026; Zhang et al., 2024). A resource-allocation account would interpret this pattern as more efficient prioritization of task-relevant information, although the present study did not directly measure resource allocation (Pearson et al., 2022).

The main effect of decision type on reaction time was not significant. Contemporary sport-decision research treats intuitive and analytical processing as a continuum, so a uniform reaction-time advantage should not be assumed (Bossard et al., 2022). Nonetheless, a descriptive tendency indicated that cognitive decisions elicited faster reactions than intuitive ones. This mismatch may result from the brief intervention duration and restricted diversity in training exercises.

The interaction between time, group, and decision type was significant. The experimental group exhibited a significant decrease in reaction time during cognitive decision tasks post-training (*p* = 0.023, *ηp*^2^ = 0.19), indicating enhanced information processing efficiency (Paradela et al., 2023). The experimental group surpassed the control group in the post-test (*p* < 0.001, *ηp*^2^ = 0.409), validating the efficacy of working memory training (Booth et al., 2023).

The primary effect of choice type was not significant (*p* = 0.27); however, the experimental group exhibited considerably quicker reaction times compared to the control group in both cognitive (*p* = 0.017) and intuitive (*p* < 0.001) situations. These findings are consistent with evidence that perceptual-cognitive training can improve decision accuracy and response time in team-sport athletes, although transfer to real competition and to officiating contexts may be smaller or uncertain (Zhu et al., 2024). It should be noted that the current intervention adopted domain-general executive function tasks (stroop, 2-back, more-odd shifting) rather than sport-specific cognitive training paradigms. According to Kalén et al. (2021), domain-general cognitive training often yields limited transfer to real-world sport performance. In the present study, significant improvement was observed only in static-image laboratory officiating tasks; we cannot directly infer that such training effects automatically transfer to dynamic, high-pressure real-game refereeing contexts. Jiang et al. (2021) also highlighted that transfer from non-sport cognitive tasks to sport-specific performance is not guaranteed. Therefore, the observed beneficial effects should be constrained to our experimental laboratory setting.

### 3.6 The effect of working memory training on working memory capacity in collegiate basketball referees

Working memory is a core cognitive function often proposed to support officiating, but direct evidence in basketball referees remains limited; recent studies instead link officiating expertise with visual search and decision accuracy and find only a subtle association between inhibitory control and disciplinary decisions (Albaladejo-García et al., 2025; Sala and Gobet, 2017; Wang et al., 2025). Recent meta-analytic and controlled evidence indicates that working memory training can improve trained or closely related tasks, but broad transfer to complex decision-making is limited or variable (Ripp et al., 2022; Syed et al., 2024).

The findings of this study indicated that following 8 weeks of working memory training, the experimental group of referees exhibited a substantial enhancement in their working memory capacity scores (*p* < 0.05), but the control group shown no significant alteration (*p* > 0.05). This study utilised the Stroop, 2-back, and More-Odd shifting tasks to assess inhibition, updating, and shifting functions, respectively, thereby perpetually engaged cognitive control and attentional regulation, which enhances information processing efficiency.

Working-memory performance can improve with training, but current evidence does not support interpreting such gains as a simple expansion of domain-general capacity beyond a fixed “7 ± 2” limit; benefits are usually largest on trained or closely related tasks (Ripp et al., 2022; Syed et al., 2024). Evidence for durable far transfer to other cognitive activities remains limited, so implications for overall officiating skill should be considered provisional (Ripp et al., 2022; Syed et al., 2024).

This study demonstrates that working memory training substantially improves the working memory capacity of collegiate basketball referees. These findings provide theoretical validation and practical ramifications for referee training, and future research should further explore the long-term consequences to enhance training programs.

Nevertheless, it is necessary to interpret our significant transfer results in light of critical meta-analyses on working-memory training. Sala and Gobet (2017) argued that far-transfer effects of working-memory training are frequently weak or non-existent. Several boundary conditions may explain why we detected significant transfer toward refereeing decision-making in our sample. First, our participants were student-referees with relatively low-to-moderate officiating experience; for populations with incomplete domain-specific expertise, domain-general cognitive capacity may place stronger constraints on task performance, creating more room for training-induced improvement (Morrison and Chein, 2011). Second, our refereeing-decision task shared high cognitive demands with working-memory measurements: both require rule retention, simultaneous information monitoring and inhibitory control. This represents near-transfer rather than broad far-transfer. Third, the 8-week protocol contained three training sessions per week, yielding relatively high total training dosage compared with many short-duration training protocols that report null transfer (Booth et al., 2023). We do not claim unlimited broad far-transfer; improvements were restricted to laboratory-based officiating-relevant cognitive tasks in this specific sample.

## 4 Conclusion

1. In comparison to the control group, an 8-week working memory training regimen markedly enhanced the officiating judgement accuracy of collegiate basketball referees, with cognitive decision-making task accuracy significantly surpassing that of intuitive decision-making tasks. These medium-magnitude improvements are restricted to referees with limited officiating experience and should not be generalized to professional elite referees.
2. In comparison to the control group, 8 weeks of working memory training markedly decreased the decision reaction time of collegiate basketball referees. This training-related benefit is sample-specific and may not hold for highly-experienced professional referees.
3. Working memory training significantly improved the working memory capacity of collegiate basketball referees. We caution against generalizing this finding to referee groups with higher officiating expertise.

### 4.1 Limitations and future research directions

This study has several limitations. First, the sample size was relatively small (*N* = 28), and participants were all collegiate referees from a single university in China, which limits the generalizability of the findings to broader or higher-level referee populations. Second, the officiating decision-making task used static images rather than dynamic laboratory simulated officiating tasks based on static images, reducing ecological validity. Third, the intervention lasted only 8 weeks without follow-up assessments, leaving the long-term sustainability of training effects unknown. Fourth, the exclusion criteria in the Methods section were incompletely reported, affecting transparency. Future research should recruit larger and more diverse samples (e.g., professional referees), incorporate longitudinal follow-ups to assess durability, develop more ecologically valid video-based or virtual reality decision-making tasks, and examine transfer effects to actual game performance. Additionally, future studies may explore optimal training dosages and combine working memory training with other cognitive or perceptual interventions.

Second, the officiating decision-making task used static images rather than dynamic game scenarios, reducing ecological validity. Moreover, our working-memory intervention relied on domain-general cognitive tasks instead of basketball-referee-specific training materials. Previous meta-analyses indicate that cross-domain transfer from general cognitive training to real-sport performance may be weak or absent (Kalén et al., 2021; Jiang et al., 2021). Therefore, generalization to real-competition officiating should be made with great caution. Future work could integrate sport-embedded working-memory training stimuli together with video-based or VR dynamic decision-making tasks.

our control group was a no-training blank control rather than an active placebo cognitive control group. Therefore, we cannot fully disentangle true working-memory-training-specific effects from potential Hawthorne effects, participant expectation effects, or pure repeated-testing practice effects. The significant time-by-group interaction supports differential change between groups, yet active placebo control is strongly recommended for future intervention studies to isolate training-specific effects.

## Data Availability

The original contributions presented in the study are included in the article/supplementary material, further inquiries can be directed to the corresponding author.
